# Anti-NGF treatment worsens subchondral bone and cartilage measures while improving symptoms in floor-housed rabbits with osteoarthritis

**DOI:** 10.3389/fphys.2023.1201328

**Published:** 2023-06-26

**Authors:** Stephanie Menges, Martin Michaelis, Kerstin Kleinschmidt-Dörr

**Affiliations:** ^1^ Merck Healthcare KGaA, Darmstadt, Germany; ^2^ Merck KGaA, Darmstadt, Germany

**Keywords:** osteoarthritis, subchondral bone, rabbits, anti-NGF, group housing

## Abstract

**Objective:** Osteoarthritis (OA) is a common joint disorder often affecting the knee. It is characterized by alterations of various joint tissues including subchondral bone and by chronic pain. Anti-nerve growth factor (NGF) antibodies have demonstrated improvement in pain associated with OA in phase 3 clinical trials but have not been approved due to an increased risk of developing rapidly progressive OA. The aim of this study was to investigate effects of systemic anti-NGF-treatment on structure and symptoms in rabbits with surgically induced joint instability.

**Methods:** This was elicited by anterior cruciate ligament transection and partial resection of the medial meniscus in right knee of 63 female rabbits, housed altogether in a 56 m^2^ floor husbandry. Rabbits received either 0.1, 1 or 3 mg/kg anti-NGF antibody intra-venously at weeks 1, 5 and 14 after surgery or vehicle. During in-life phase, static incapacitance tests were performed and joint diameter was measured. Following necropsy, gross morphological scoring and micro-computed tomography analysis of subchondral bone and cartilage were performed.

**Results:** After surgery, rabbits unloaded operated joints, which was improved with 0.3 and 3 mg/kg anti-NGF compared to vehicle injection during the first half of the study. The diameter of operated knee joints increased over contralateral measures. This increase was bigger in anti-NGF treated rabbits beginning 2 weeks after the first IV injection and became dose-dependent and more pronounced with time. In the 3 mg/kg anti-NGF group, the bone volume fraction and trabecular thickness increased in the medio-femoral region of operated joints compared to contralateral and to vehicle-treated animals, while cartilage volume and to a lesser extent thickness decreased. Enlarged bony areas were found in right medio-femoral cartilage surfaces of animals receiving 1 and 3 mg/kg anti-NGF. Alterations of all structural parameters were particularly distinct in a subgroup of three rabbits, which also exhibited more prominent symptomatic improvement.

**Conclusion:** This study showed that anti-NGF administration exerted negative impact on structure in destabilized joints of rabbits, while pain-induced unloading of joints was improved. Our findings open up the possibility to better understand the effects of systemic anti-NGF, particularly on subchondral bone, and thus the occurrence of rapidly progressive OA in patients.

## 1 Introduction

Osteoarthritis (OA) is the most common disease of the human musculoskeletal system and is characterized by progressive degradation of various joint tissues including subchondral bone, cartilage and by chronic pain (McCune et al., 1990; Naredo et al., 2005).

While there are currently no therapeutic approaches that have been shown to slow down or reverse disease progression, therapy focuses on pain relief and preservation of joint function. Nonsteroidal anti-inflammatory drugs (NSAIDs) have been the mainstay of treatment for more than a century. A large number of OA patients are unable to take NSAIDs due to intolerable side effects related to various conditions (Miller et al., 2017b), while other analgesics like opiates or tramadol are leading to increased morbidity, particularly in elderly patients (Malfait and Block, 2015). Over the years of understanding OA pathophysiology regarding its pain mechanisms, the neurogenic origin and responsiveness to neutralization of particular neurotransmitters became apparent (Malfait and Schnitzer, 2013; Miller et al., 2014). Besides its role as a growth factor for nerve cells, nerve growth factor (NGF) was identified as a key mediator of acute and chronic pain (Lewin et al., 1993; Mizumura and Murase, 2015; Denk et al., 2017). It is released by immune cells as part of the inflammatory response following peripheral injury. NGF binds with high affinity to a member of the tropomyosin-related kinase (Trk) family (TrkA) on the neuronal cell surface and immune cells, which results in activation of various pathways. Up to now, the role of NGF in chronic pain signaling is not fully understood. It is believed, that as a short-term action NGF not only sensitizes the nociceptor but also in the long term stimulates survival of neurons, induces nerve sprouting or growth (Bothwell, 2016; Denk et al., 2017; Schmelz et al., 2019). In addition, binding on TrkA on immune cells induces the release of inflammatory mediators like histamine, PGE2, 5-HT, serotonin and also NGF itself, which further contributes to sensitization (Horigome et al., 1993; Marshall et al., 1999; Mantyh et al., 2011). Those pronociceptive actions of NGF, like increase in nociceptive signaling through the dorsal horn and supraspinal structures, contribute to its role as one of the key mediators in pain. As a consequence, targeting the pro-algesic effects elicited by NGF via antagonization turned out to be an attractive strategy for pain relief (Mantyh et al., 2011) and were therefore of particular interest in the context of OA. Other emerging disease-modifying approaches in preclinical and clinical development showing beneficial potential in OA include treatments with, e.g., fibroblast growth factors (e.g., FGF18), bone morphogenetic proteins (e.g., BMP7), growth and differentiation factors (e.g., GDF5), extracellular matrix phosphoglycoprotein (MEPE), or anti-inflammatory drugs, like interleukin inhibitors or inverse agonists of retinoic acid-related orphan receptor alpha (RORalpha) (Zhang et al., 2019; Gigout et al., 2022). In contrast to anti-NGF inhibitors, they target the mechanistic pathophysiology of osteoarthritis by regulating catabolic and anabolic processes in the cartilage and bone or inflammatory responses in the synovium, but do not address the modulation of acute and chronic pain by effecting nociceptor sensitization. Anti-NGF antibodies have demonstrated promising results in alleviating pain associated with OA (Miller et al., 2014; Schnitzer and Marks, 2015; Kan et al., 2016; Chen et al., 2017) but at the same time clinical trials have revealed serious adverse effects including rapidly progressive OA (RPOA) and osteonecrosis (Hochberg, 2015; Maloney et al., 2016; Lane and Corr, 2017). Therefore, United States and European health authorities have not yet approved this treatment option (EMA, 2021; FDA, 2021).

Before its application in clinical trials, nerve growth factor blockade with antibodies showed improved symptomatic measures in various preclinical OA-studies in rodents (McNamee et al., 2010; Bryden et al., 2015; Flannery et al., 2015; Ishikawa et al., 2015; Kc et al., 2016; Nwosu et al., 2016; Xu et al., 2016; LaBranche et al., 2017), cats (Gruen et al., 2016) and dogs (Lascelles et al., 2015). Despite the fact, that rabbits are frequently used in OA-research studies since decades (Hulth et al., 1970; Oegema and Visco, 2020), to our knowledge no rabbit data have been published on anti-NGF treatment in OA-models by now.

In research, rabbits were and are traditionally housed in small cages with limited degree of locomotion. To allow species-appropriate movement patterns (Laverty et al., 2010) and thereby to enhance the translational value of readouts, we implemented a floor housing for up to 65 rabbits (Gigout et al., 2022). The purpose of the current study was to evaluate the effects of systemic anti-NGF treatment in a rabbit model of surgically induced joint instability, focusing on structural readouts like subchondral bone and cartilage and behavioral measures.

## 2 Materials and methods

### 2.1 Animals and housing conditions

The animal experiment was designed and performed in accordance with the German animal welfare act and approved by the regional authority of Darmstadt, Hesse, Germany (approval number DA 4/1009).

**FIGURE 1:**
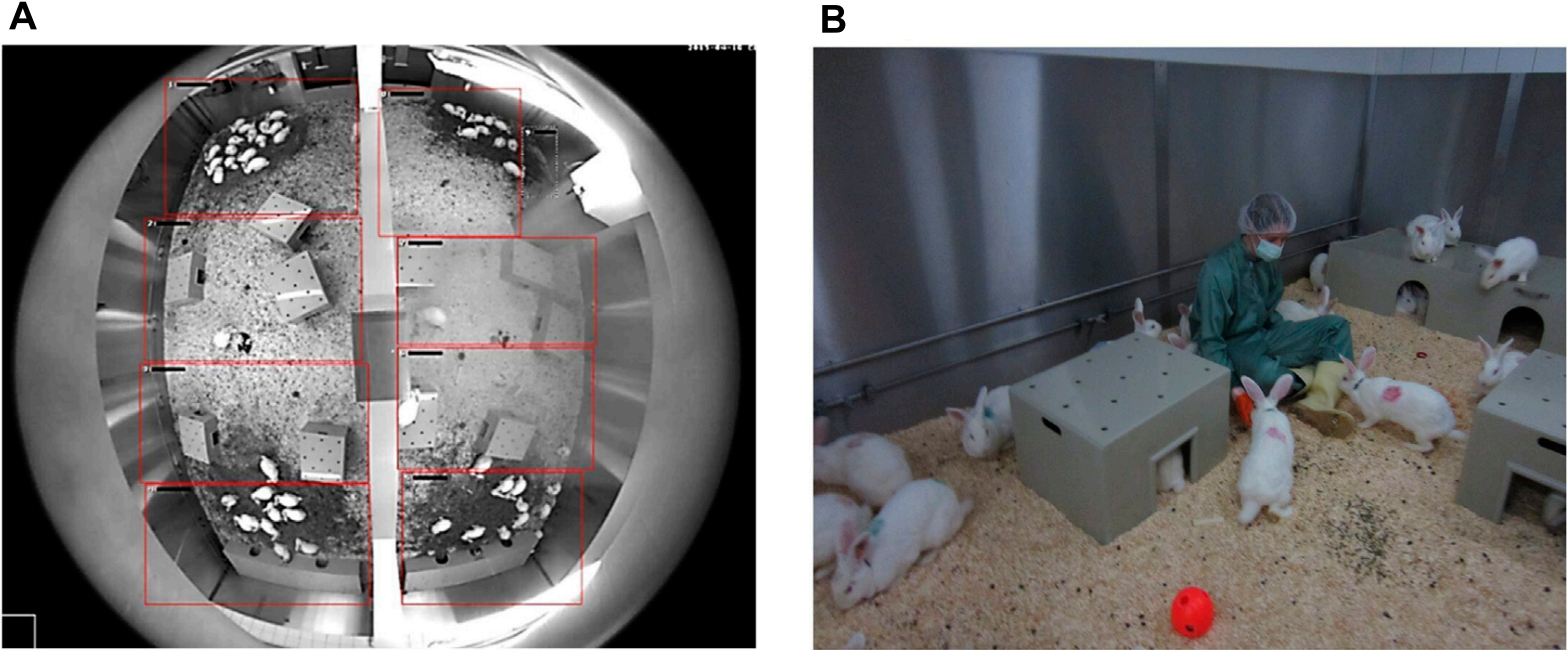
**(A)** Top view of the housing via fisheye-camera: A half-height wall (here: vertical in the middle) divides the husbandry in two smaller rooms. Below two large shelters are placed, smaller shelters are distributed evenly in the housing. Rabbits gather at the haystacks. **(B)** Rabbits with color-marks on the fur after surgery and collars for activity tracking. The circular waterline with drinking valves is visible in the back. Enrichment (wooden gnaw sticks, balls, etc.) can be found on the floor. Animals show curious behavior towards humans in the housing.

65 Female New Zealand White rabbits Hsdlf:NZW, 8 weeks of age, were purchased from Envigo U.K. immediately after weaning and housed together in a 56 m^2^ floor pen (Figure 1A), allowing the animals unlimited running, jumping, and socializing for 6 months prior to the start of the study at 8 months of age.

The housing conditions fulfilled provisions of the Directive 2010/63/EU (DIRECTIVE, 2010/63/EU, 2010) and the Guide for the Care and Use of Laboratory Animals (National Research Counsil, 2011). Dry-bulb temperatures were chosen from 15°C–21°C, with lower and upper critical temperatures defining the boundaries for rabbit housing (Gonzalez et al., 1971). Ranges for relative humidity were defined from 45%–65% and air changes remained around 4000 m³ per hour with 2 m/s in the room and <0.5 m/s at the animals. The facility operated on overpressure. Light and dark phase remained equal with light switched on from 6 a.m.–6 p.m.

Animals received *γ*-radiated hay *ad libitum* (H3279-622, ssniff^®^, Soest, Germany) and a restricted amount of diet pellets (K-H V2333, ssniff^®^) on a daily basis. The amount of food pellets scattered on the floor of the housing was always calculated to provide two-third of the energy needed (Szendrő et al., 2011) and over the time adjusted to the increasing average body weight of the colony. Water was provided by a custom-built circumferential drinking line with 16 valves connected to the tap water system. For enrichment, shelter, and toys (wooden gnaw sticks, different enrichment toys from Bio-Serv^®^, Flemington, USA) were provided (Figure 1B).

Upon arrival, animals were marked with a radiofrequency identification chip (Slim T-SL Microchip 10.9 × 1.6 mm and 2.0 mm needle, Datamars, Bedano, Switzerland) in the lateral region of the neck. Health monitoring was performed according to FELASA-guidelines.

We focused on female rabbits because group housing of a comparable number of male rabbits would pose a significant risk of injury to the animals. In addition, mixed group housing of female and male rabbits for 12 months was not feasible due to the sexual maturity of the animals during this time.

Exclusion criteria were applied, when, e.g., an animal had to be euthanized due to animal welfare reasons by defined humane endpoints. Those criteria were documented in the animal proposal and approved by the authorities. One rabbit had to be euthanized 2 days after delivery at young age due to paraplegia and one rabbit died during surgery in anesthesia (0.3 mg/kg anti-NGF group, *n* = 11 reduced to *n* = 10).

Every procedure was performed in a blinded way to ensure observer-independent readouts.

### 2.2 Surgically induced OA, joint diameter, and injections

Anesthesia, surgery, and injections were in accordance with GV-SOLAS recommendations in effect at the time. Rabbits were normalized to a mean body weight when allocated to groups, to ensure even body weight distribution. Groups were divided into vehicle (*n* = 16), research compound (*n* = 16; data not shown in this paper), 0.3 mg/kg anti-NGF (*n* = 10), 1 mg/kg anti-NGF (*n* = 10) and 3 mg/kg anti-NGF (*n* = 11). Group sizes of vehicle and research compound were calculated via a sample size calculator based on former internal study results of incapacitance (power 0.8, alpha 0.05), while anti-NGF-antibody was tested in 3 ascending doses to investigate the effects of systemic treatment. Doses of anti-NGF were selected based on available literature data from other species (Miller et al., 2017a) and due to results from a former study (Gigout et al., 2022). Data obtained from rabbits who received research compound were considered for statistics where available (joint diameter, static incapacitance and body weight); structural data from research compound were not generated and analyzed due to project closure. Surgery was stretched over 2 weeks (as maximum number of surgeries was *n* = 8/day), starting with the heaviest animals. On the day of surgery, operations were performed on animals from all groups in a randomly allocated order (via randomizer.org), while the two surgeons were blinded to group allocation.

At least 30 minutes prior to surgery, rabbits received Temgesic^®^ 0.01 mg/kg SC (Indivor Dublin, Ireland), while Robinul^®^ 0.1 mg/kg SC (Riemser, Greifswald, Germany) and Domitor^®^ 0.2 mg/mL IM (Vetoquinol, Ismaning, Germany) were administered immediately before surgery. Isoflurane 2%–4% was inhaled in 1.0 L/min carbogen to induce and maintain anesthesia via a face mask. Anaesthetized rabbits were positioned on a heating pad at 38°C. The right knee joint was shaved and disinfected with Cutasept®F (Hartmann, Heidenheim, Germany) and kept wet with Braunol^®^ (B. Braun, Melsungen, Germany) on sterile gauze swabs. After transfer to the surgery room, rabbits were slowly infused IV with 50 mL of 5% glucose solution per animal (B. Braun) via a catheter into the marginal ear vein during the course of surgery (approx. 20–30 min). Intraoperative monitoring was performed via pulse oximetry clipped on the tongue (Palm SAT^®^ 2500A VET Pulsoximeter, NONIN Medical, Amsterdam, Netherlands) and by a temperature probe. The animal was covered with sterile surgical drape (Raucodrape, Lohmann and Rauscher, Rengsdorf, Germany).

Following reflex testing, skin, muscle, and capsule were opened under sterile conditions using scalpel and scissors. The fat pad was clamped aside to expose the anterior cruciate ligament (ACL). Transection of the ligament was performed using a small ear hook and a scalpel. Afterwards, the anterior horn of the meniscus was uncovered and detached from the meniscotibial ligament. The anterior half of the meniscus was resected (pMx) with small scissor under fixation with a clamp. The joint was irrigated with 5 mL of sterile physiological saline solution (B. Braun) and the capsule and skin were closed in three layers using resorbable suture material (Vicryl Plus 5–0, FS-2 needle, Ethicon, Raritan, USA).

After surgery, rabbits were positioned single-housed in a R-Suite Rack (Tecniplast, Varese, Italy) on hay and covered with an undersheet to prevent cooling. Post-operative monitoring was secured via pulse oximetry via the tongue. Immediately, Antisedan^®^ 1 mg/kg IM (Vetoquinol) was administered to antagonize the sedative effect of Domitor^®^ (around 1.5 h after Domitor^®^ injection) and allowed rapid recovery of the rabbits. Besides the breeders’ ear tattoo and chip identification, animals were color-marked on their fur according to their day of surgery. To ensure post-surgical analgesia, Metacam^®^ 0.2 mg/kg SC (Boehringer Ingelheim, Ingelheim, Germany) was injected for 3 consecutive days starting at the day of surgery. When the animals were fully awake (approximately 4–5 h after surgery), they were returned to the group housing and allowed to move freely in the housing pen until the end of the study. Post-surgical health monitoring and scoring was performed daily for 1 week, afterwards individual health scoring was performed on a weekly base. Body weight was assessed weekly and analyzed as delta to week 1 (see Supplementary Figure S1).

The diameter of left and right knee joints was measured before surgery and in weeks 1, 3, 5, 7, 10, 14 and 16 post surgery. Measurements were taken with a digital and calibrated caliper (0–150 mmm, VWR, Darmstadt, Germany). Legs had to be shaved and measurements were performed on awake animals before injections. Joint diameters were normalized to week 1 and the % increase of the diameter of the operated (right) knee joint compared to contralateral one was calculated.

Vehicle (and research compound) were injected IA into the right knee joint in weeks 1, 5 and 14 after surgery, while anti-NGF groups received vehicle IA, as well. Anti-NGF was injected IV according to the same time schedule, while the vehicle (and research compound) group received equal volume of sterile saline solution IV. For IA injections, rabbits were anesthetized with isoflurane 2%–4% in 1.0 L/min carbogen and joints were shaved, disinfected, and gently flexed. Injections were made into the right knee joint at a 90° angle to the knee surface using a 26G cannula and afterwards flexed and stretched for three times to allow even distribution of the injected volume of 200 µL. IV injections with 5 mL/kg were performed via the marginal ear vein through a 22G braunule during the same anesthesia. Timepoints for injection were chosen based on internal results with the research compound in former studies and with the aim of analyzing the duration of action for this research compound.

Sixteen weeks after surgery, all rabbits were euthanized under deep isoflurane narcosis with Narcoren^®^ 160 mg/kg (Boehringer Ingelheim) via administration through the marginal ear vein, and knee joints were prepared for gross-morphological investigation and imaging.

### 2.3 Static incapacitance

Static incapacitance test was performed contact-free to ensure observer independence with a custom-made device. Details regarding incapacitance method can be found in the supplementary methods of (Gigout et al., 2022). Incapacitance was measured once prior to surgery for baseline assessment and weekly from week 1–16, always before injection. Weight bearing was calculated from the final values as follows:

$$weight\;bearing\;right\;\left[\%\right]=\left(\frac{mean\;raw\;data\;right}{mean\;raw\;data\;right+mean\;raw\;data\;left}\right)\times 100$$

Values at time course graph were normalized to week 1 and depicted as % improvement. Due to staff illness, measurements in weeks 8 and 10 were incomplete and therefore excluded from analysis in total.

### 2.4 Gross morphology of cartilage surfaces

After euthanasia, the knee joints were dissected and cleaned from soft tissue. The articular surfaces of the femora were stained with toluidine blue (0.05%) for 30 s at room temperature, washed briefly with demineralized water, and air-dried for 15–20 min. Afterwards, the samples were dipped in black ink (Higgins black India ink, Chartpak Inc.) for 1 s, removed from the solution for 3 s, and then washed with tap water. After an additional air-drying period of 15 min, the surfaces were imaged using a Discovery V12 macroscope (Carl Zeiss Microscopy, Jena, Deutschland) and photographed using an Axiocam HRC camera and appropriate software AxioVision 4.8.2 (Carl Zeiss Microscopy). 10–20 individual images were acquired in a Z-stack and combined to the final image for the scoring. The medial joint surfaces of the right femora were scored individually, based on a modified Mankin score (Ren et al., 2018). Increasing grades indicated higher damage: 0 = smooth with blue color, healthy; 1 = irregularities in black, no shiny blue color; 2 = thin cartilage, pale color but no white bone; 3 = lilac color, area of white bone <50% of surface; 4 = large bony areas >50% of surface.

After gross morphology, knee joints were fixed in 4% paraformaldehyde (PFA, Merck, Darmstadt, Germany; in 1x phosphate buffered saline (PBS) pH 7.4, Gibco, Waltham, USA) for at least 7 days to achieve full fixation for imaging.

### 2.5 Micro-computed tomography

Prior to micro-computed tomography (micro-CT imaging), the joints were rinsed in 1x PBS for 2 h to remove residual PFA. Afterwards, a little plastic container was filled with a nonabsorbable contrast agent with a radiodensity comparable to that of subchondral bone (Moltofill, Akzo Nobel, Cologne, Germany) to allow cartilage segmentation. Femora were inserted each into a container, until the cartilage surface was completely covered with Moltofill.

Specimens were scanned using a SkyScan 1176 micro-CT scanner (Bruker, Kontich, Belgium) with an x-ray source of 90 kV/278 μA, a pixel size of 17.60 µm and a 0.1 mm copper filter. Cross-sectional slices were generated using Nrecon software (version 1.7.4.6, Bruker) with beam hardening at 40%, smoothing at level 5, and ring artifact corrections at level 7 applied and using defined threshold values (lower grey = 70, upper grey = 184) to segment radiolucent cartilage from radiopaque contrast agent and bone. All data sets were adjusted to anatomical markers with DataViewer software (version 1.5.4, Bruker) in the same manner to ensure uniform analysis of the datasets. Automated three-dimensional analysis was performed using CTAn software (version 1.17.7.2, Bruker) to evaluate bone volume per tissue volume (BV/TV, TV was equivalent to total volume of interest) and trabecular thickness (Tb.Th) respectively cartilage volume (CV) and cartilage thickness (C.Th). Exactly equal cylindric volumes of interest (VOI) in the center of the weight-bearing region of the medial and lateral femoral condyles of rabbits were applied (bone analysis in trabecular region: VOI, 200 pixels in diameter, 101 images inside VOI; cartilage analysis: VOI, 600 pixels in diameter, 71 images inside VOI, see Figure 5E). Individual data points were normalized to the contralateral values of each animal and depicted as % contralateral in the plots.

### 2.6 Statistical analysis

Statistical analysis was performed with GraphPad Prism version 7.03. The respective tests used are indicated in the figure legends.

All data are presented as mean ± SEM. XY-graphs were used for body weight gain and joint diameter. Weight bearing, imaging data from bone and cartilage and gross morphology scoring were expressed as scatter dot plots.

Outlier testing was performed using the ROUT method provided by GraphPad Prism (coefficient [Q] = 1%; identifies outliers from nonlinear regression). Individual data were excluded if the ROUT method identified a particular data point as an outlier.

Before applying statistical tests, data were checked for normal (Gaussian) distribution with Shapiro-Wilk normality test (significance level (alpha) of 0.05).

Multiple comparisons were conducted with comparing the mean of each column with the mean of every other column.

## 3 Results

### 3.1 Static incapacitance

**FIGURE 2:**
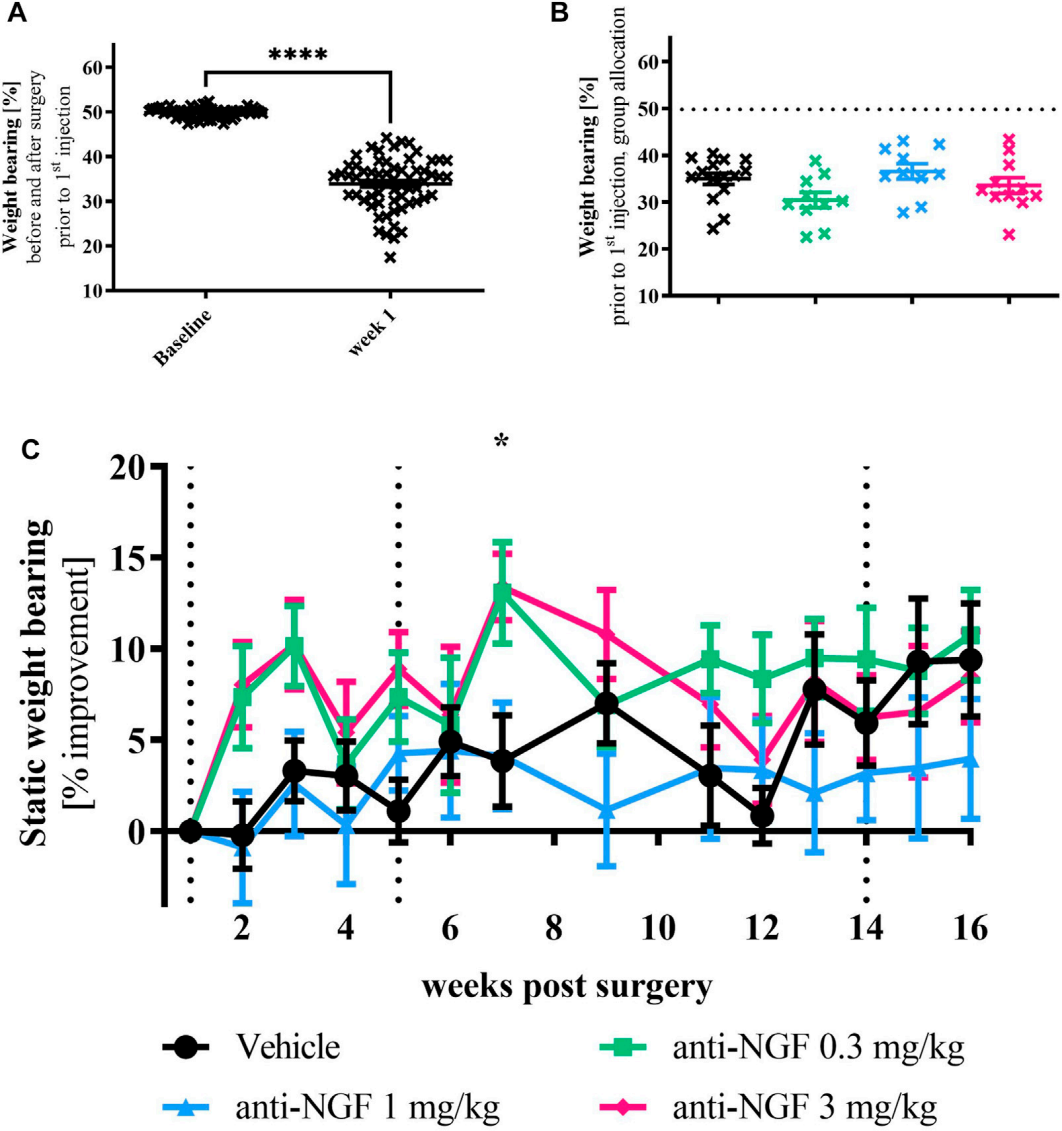
**(A)** Weight bearing baseline vs post-surgery: rabbits were measured for baseline before surgery (left) showing even weight distribution and 1 week after surgery (right); *n* = 63; mean ± SEM; no outliers via ROUT method detected; data passed Shaprio-Wilk test for normal distribution; results of paired *t*-test: ****p < 0.0001. **(B)** Weight bearing data of all animals after surgery but before treatment divided into their respective groups without group names shown; *n* = 10–15; mean ± SEM; dotted horizontal line indicates mean of all animals at baseline; one outlier via ROUT method detected and excluded from analysis (Vehicle 17.44%); data did not pass Shaprio-Wilk test for normal distribution; no significant differences in Kruskal–Wallis test with Dunn’s multiple comparison test. **(C)** Weight bearing time course; *n* = 10–16; mean ± SEM; data are normalized to week 1 before injection; dotted black line marks timepoint of injection of all groups; no outliers via ROUT method detected; data passed Shaprio-Wilk test for normal distribution; results of 2way ANOVA with Dunnett multiple comparison test: week 7 *p < 0.05 Vehicle vs anti-NGF 3 mg/kg.

Before surgery, all rabbits showed an equal distribution of weight to both hind legs (Figure 2A). Seven days after instability surgery on the right leg, weight bearing on the operated side was strongly reduced in most animals (*p* < 0.0001).

When splitting the post-operative weight bearing results from week 1 before the first injection into the different treatment groups, a heterogeneous distribution of the data with slight differences in means could be observed (Figure 2B).

During the first half of the experiment, unloading of operated joints was moderately improved with 0.3 and 3 mg/kg anti-NGF compared to vehicle injection (Figure 2C), reaching significant improvement via 3 mg/kg anti-NGF-treatment in week 7 (*p* = 0.0383). At later timepoints, no significant discrimination of symptomatic anti-NGF effects was possible. Among rabbits treated with 3 mg/kg anti-NGF, higher improvement in static weight bearing was particularly distinct in a subgroup of three rabbits (see Supplementary Figure S2).

Additional analysis revealed, that incapacitance data (overall calculated as area under the curve and week 16) did not correlate with bone imaging data.

### 3.2 Joint diameter

**FIGURE 3:**
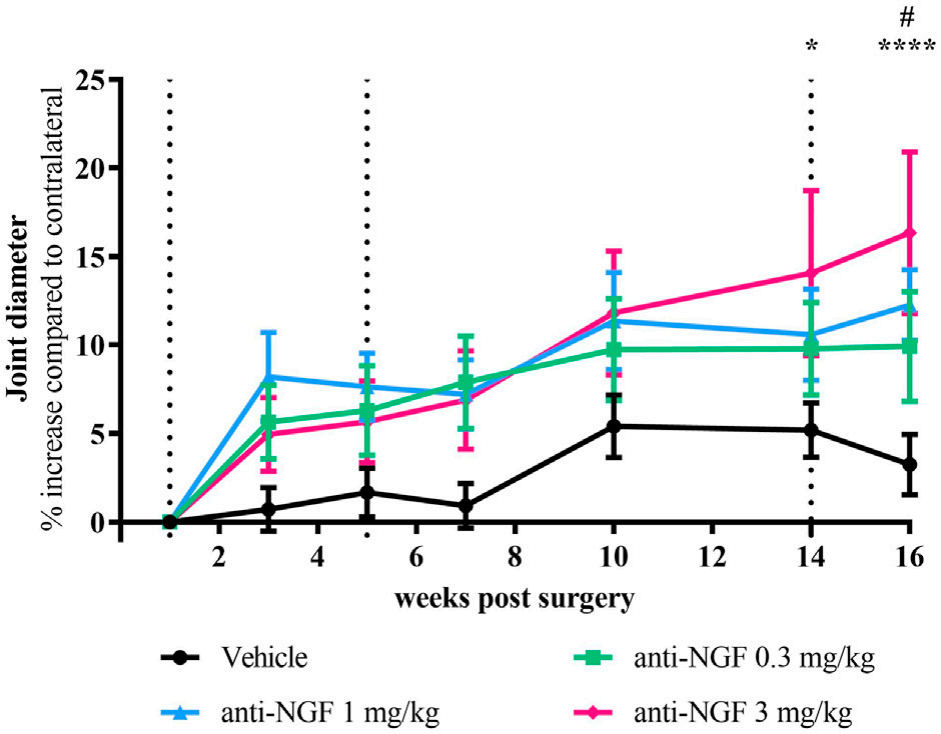
Time course of joint diameter; *n* = 10–16; mean ± SEM; data are normalized to week 1 before injection; dotted black line marks timepoint of injection of all groups; no outliers via ROUT method detected; data passed Shaprio-Wilk test for normal distribution; results of 2way ANOVA with Dunnett multiple comparison test: week 14 *p < 0.05 Vehicle vs anti-NGF 3 mg/kg; week 16 #*p* < 0.05 Vehicle vs anti-NGF 1 mg/kg, ****p < 0.0001 Vehicle vs anti-NGF 3 mg/kg.

The diameter of operated knee joints was larger than of contralateral joints in all treatment groups (Figure 3). Already 2 weeks after the first IV injection, this increase compared to contralateral was more pronounced in anti-NGF-treated rabbits than in the vehicle treated group (Figure 3). Joint broadening continued to progress over time with dose-dependent trends towards the end of the study after 16 weeks. Increase in joint diameter compared to contralateral was significantly higher in the 3 mg/kg anti-NGF group than in the vehicle group at 14 weeks (*p* = 0.0154) and 16 weeks (*p* < 0.0001), while in the 1 mg/kg of anti-NGF group only at 16 weeks (*p* = 0.0176) when compared to vehicle. 0.3 mg/kg anti-NGF contributed to joint broadening without reaching significant levels. Effects on joint diameter gain in the subgroup of three rabbits after treatment with 3 mg/kg of anti-NGF was significantly higher compared to the rest of this group containing 8 rabbits (see Supplementary Figure S3), especially in late stages of treatment from weeks 10–16 (week 10 **p < 0.01; week 14 ***p < 0.001; week 16 ***p < 0.001).

### 3.3 Gross morphology of cartilage surfaces

**FIGURE 4:**
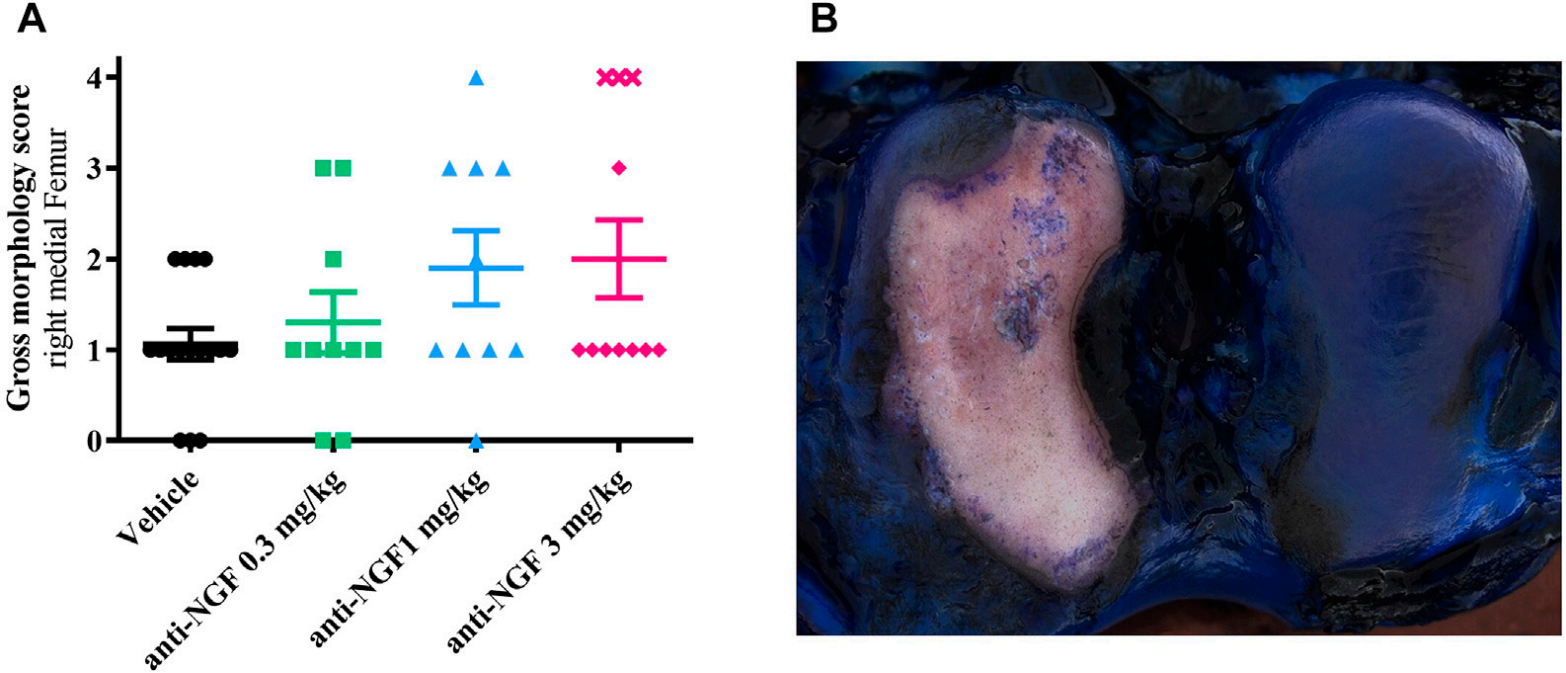
**(A)** Gross morphology score from medial right femur of all groups; *n* = 10–16; mean ± SEM; no outliers via ROUT method detected; data did not pass Shaprio-Wilk test for normal distribution; no significant differences in Kruskal–Wallis test with Dunn’s multiple comparison test. **(B)** Gross morphology picture of right femur after instability surgery and systemic anti-NGF treatment with 3 mg/kg IV; score 4 (medial) and score 1 (lateral).

Reflecting the findings from cartilage analysis via micro-CT, gross-morphological scoring of the cartilage surfaces from right medial femora revealed enlarged bony areas (Figure 4B) in animals receiving 1 and 3 mg/kg anti-NGF (Figure 4A).

Again, as previously observed for bone parameters, highest scores were detected in the subgroup of three rabbits treated with 3 mg/kg anti-NGF (marked as cross symbols, Figure 4A).

A total of six rabbits (three of vehicle group, two of 0.3 mg/kg anti-NGF group and one of 1 mg/kg anti-NGF group) scored zero, with smooth cartilage surfaces with blue color on their medial femoral regions and no apparent cartilage defects in the gross morphological evaluation.

### 3.4 Micro-CT of bone and cartilage

**FIGURE 5:**
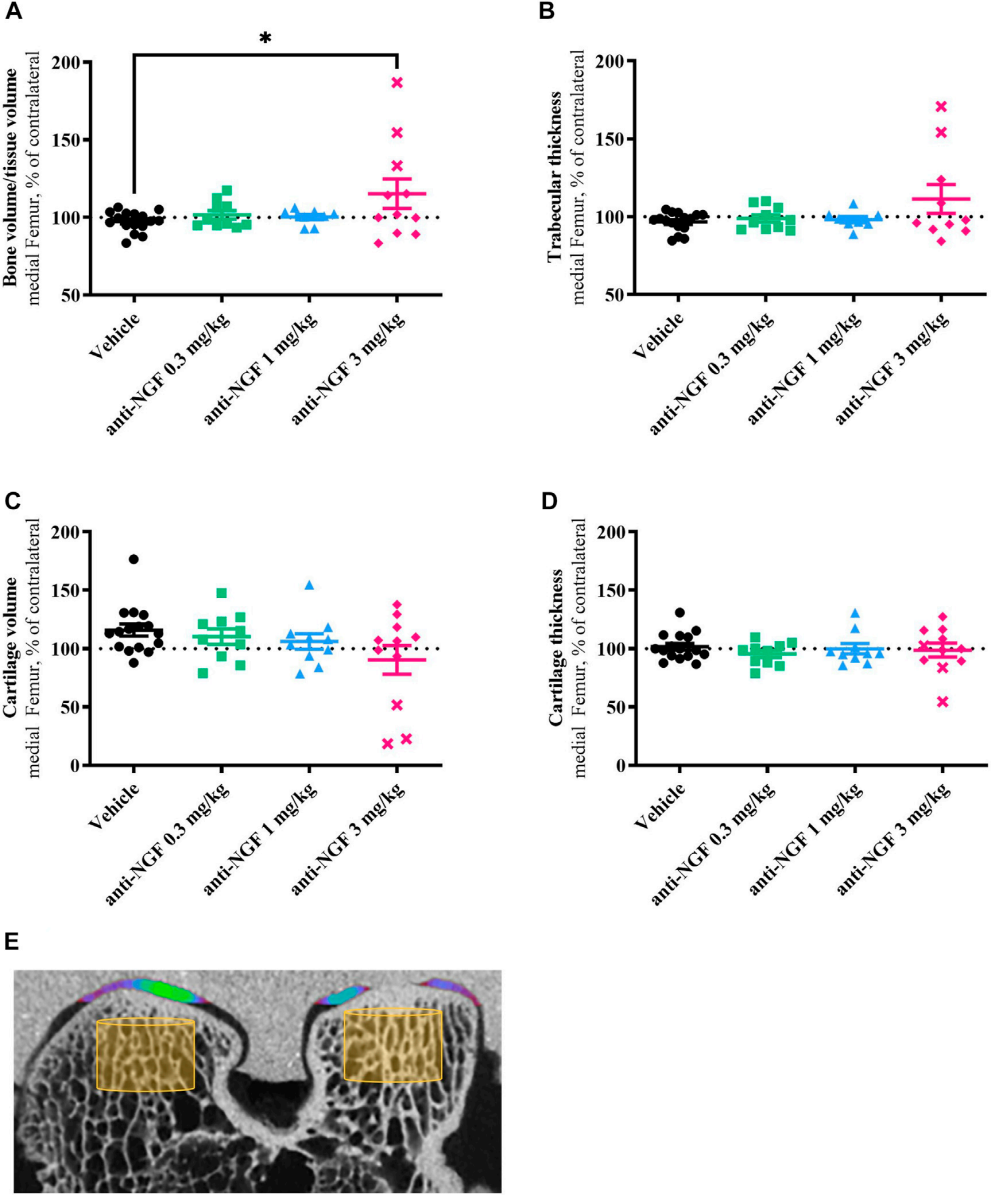
**(A)** Bone volume fraction in comparison to the medial contralateral part; n = 8–16; mean ± SEM; dotted horizontal line indicates 100 %; two outliers via ROUT method detected and excluded from analysis (anti-NGF 1 mg/kg: 145.32%, 166.79%); data passed Shaprio-Wilk test for normal distribution; result of 1way ANOVA with Tukey multiple comparison test: *p < 0.05 Vehicle vs anti-NGF 3 mg/kg. **(B)** Trabecular thickness in comparison to the medial contralateral part; *n* = 8–16; mean ± SEM; dotted horizontal line indicates 100%; three outliers via ROUT method detected and excluded from analysis (anti-NGF 1 mg/kg: 149.60%, 246.81%; anti-NGF 3 mg/kg: 315.79%); data did not pass Shaprio-Wilk test for normal distribution; no significant differences in Kruskal–Wallis test with Dunn’s multiple comparison test. **(C)** Cartilage volume in comparison to the medial contralateral part; *n* = 10–16; mean ± SEM; dotted horizontal line indicates 100%; no outliers via ROUT method detected; data did not pass Shaprio-Wilk test for normal distribution; no significant differences in Kruskal–Wallis test with Dunn’s multiple comparison test. **(D)** Cartilage thickness in comparison to the medial contralateral part; *n* = 10–16; mean ± SEM; dotted horizontal line indicates 100%; no outliers via ROUT method detected; data did not pass Shaprio-Wilk test for normal distribution; no significant differences in Kruskal–Wallis test with Dunn’s multiple comparison test. **(E)** Micro-CT image example of right femur with volume of interest for bone analysis (yellow cylinder) and color-coded cartilage thickness revealing a cartilage defect on medial condyle.

By micro-CT analysis after 16 weeks, we found significantly increased amounts of mineralized tissue defined as BV/TV in the right medial femoral compartment of rabbits treated with 3 mg/kg anti-NGF when compared to vehicle (*p* = 0.0432) and when compared to the contralateral joint part (Figure 5A). 0.3 mg/kg and 1 mg/kg of anti-NGF did not affect BV/TV in the right medial femur when compared to vehicle or their contralateral joint compartment after 16 weeks.

Similar effects were observed in Tb.Th data, i.e., increased thickness of subchondral trabecular structures in the operated medial femur of the high-dose anti-NGF group when compared to vehicle and contralateral joint (Figure 5B). Trabecular thickness in the medial part did not change under vehicle, low- and medium-dose anti-NGF treatment.

Concerning BV/TV and Tb.Th, rabbits treated with 3 mg/kg anti-NGF showed high intragroup variability. Both parameters were particularly distinct in the subgroup of three rabbits; data from these three rabbits are represented separately by cross symbols (Figures 5A–D).

Cartilage volume (Figure 5C), measured as radiolucent tissue in the analyzed region, more than cartilage thickness (Figure 5D) slightly decreased in the 3 mg/kg anti-NGF group when compared to vehicle in the medio-femoral region of operated knees with regards to the contralateral areas. Rabbits receiving vehicle, 0.3 mg/kg, and 1 mg/kg anti-NGF revealed no clear changes in cartilage volume and thickness in medial femur regions. Cartilage volume in vehicle and low anti-NGF dose group showed tendencies to be enlarged compared to their contralateral regions.

As already observed in bone parameters, distinct effects in the same subgroup of three rabbits could be detected regarding cartilage volume, as well.

No differences in BV/TV, Tb.Th, CV, and C.Th were observed in lateral compartments of operated vs unoperated legs in all groups (data not shown).

When comparing the effects on increased BV/TV and loss in CV, treatment with 3 mg/kg anti-NGF reveals significant correlation (*p* < 0.001) between both structural parameters.

## 4 Discussion

As a musculoskeletal disease, the characteristics of OA strongly depend on the actual use of the affected joint. Despite the known relationship between OA and physical activity, most rabbit models of OA are typically conducted in animals housed in cages. In comparison to wild rabbit habitat sizes of approximately 50 m^2^ (Surridge et al., 1999), the largest European standard rabbit enclosure size of 0.54 m^2^ (DIRECTIVE, 2010/63/EU, 2010) represents only 1% of their natural home range size. Traditional housing in individual cages with limited space prevents normal hopping locomotion and social interaction (Podberscek et al., 1991). Of particular note, the length of a single physiological step of a rabbit ranges from 35 cm to 200 cm, while the longest jump of a rabbit measured was even 301 cm (Lumpkin and Seidensticker, 2011), whereas the standard rabbit cage has a maximum width of less than 100 cm. By providing a total of 56 m^2^ we not only enhanced animal welfare aspects for the rabbits but also promoted the mechanical load burden on joints and thereby improved the translational ability of our OA-research. With anti-NGF antibodies causing serious adverse events observed in clinical trials (Hochberg, 2015), this study was performed to investigate the effects of systemic anti-NGF treatment in a rabbit model of surgically induced joint instability.

Micro-CT analysis revealed changes in several parameters during the development of OA in destabilized rabbit joints treated with high doses of anti-NGF. In contrast to vehicle treatment and lower anti-NGF doses, bone microarchitecture in the medial femoral area was significantly affected by high doses of 3 mg/kg anti-NGF. This was evidenced by increased bone volume and trabecular thickness, indicating features of late-stage OA, such as subchondral sclerosis (Burr and Gallant, 2012). In a comparable instability model of OA, the rat medial meniscal tear model, subchondral bone was assessed after anti-NGF treatment as well and revealed similar sclerotic results in comparison to our findings (LaBranche et al., 2017). One possible explanation for the observed enhanced OA-like alterations in subchondral bone could be the anti-NGF related analgesia and the subsequent joint overloading. On the other hand, in the monosodium iodoacetate (MIA) model of OA pain in rats, anti-NGF antibody treatment resulted in reduced pain behaviour together with a reduction in the number of subchondral osteoclasts, indicating beneficial effect on subchondral bone (Xu et al., 2016).

Within the group of rabbits who received 3 mg/kg anti-NGF there were three rabbits, where effects on bone microarchitecture were particularly distinct. Biological variation from joint to joint and even within the joint from site to site were shown to be pronounced in the rabbit (Stockwell, 1971; Pedersen et al., 2013). For this reason, group sizes should be chosen appropriately, if possible, to allow sufficient statistical analysis, especially when high intergroup variability occurs.

No differences in bone volume and trabecular thickness were observed in lateral compartments of operated vs unoperated legs in all groups, including high-dose systemic anti-NGF treatment. As the medial femoral region is presumed to be a major weight-bearing surface (Tochigi et al., 2013), especially after partial resection of the medial meniscus, one might speculate of its tendency to be more prone to progressive changes under anti-NGF treatment compared to the lateral joint part.

La Branche et al. (LaBranche et al., 2017) found significant cartilage degeneration in a rat instability model treated with anti-NGF (Tanezumab). In our study, rabbits receiving high doses of anti-NGF exhibited reduced cartilage volume, as well. Again, distinct effects could be observed in the subgroup of three rabbits.

As with micro-CT findings for cartilage, gross-morphological analysis of the medio-femoral cartilage surfaces revealed enlarged bony areas after high dose-administration of anti-NGF, too. Gross morphology is suitable for fast scoring analysis after necropsy compared to more complex methods of CT-imaging. Nevertheless, imaging can provide three-dimensional insights into the bone and cartilage structures of the joint. To investigate the cartilage quality and effects on its composition, digital histology imaging techniques should be applied in addition. In total, six animals with a gross morphological score of 0 were identified, indicating no visible cartilage damage. Deeper investigation of cartilage quality could reveal interesting insights to better understand responsiveness to instability surgery and anti-NGF-treatment. Due to project closure, no histological examination was performed.

In addition to parameter collection via imaging and gross morphology at the end of the study, longitudinal structural data were obtained via joint diameter measurements. Two weeks after the first IV injection, we observed an increase in joint diameters in operated joints of anti-NGF treated rabbits. This increase was progressed dose-dependently until the end of the study. As anti-NGF has proven analgesic effects (Chang et al., 2016), joint overloading after instability surgery may have induced exacerbation of joint damage that contributed to broadening of the joint. Furthermore, a distinct increase in joint diameter of all groups was observed after 8 weeks. At 8–10 weeks post induction, osteophyte formation is present in an osteoarthritic joint (Hayami et al., 2006; Sulaiman et al., 2021) and thereby contributes to enlarged joint diameters, as reflected in our results. Longitudinal imaging was not conducted in our experiment. However, a final micro-CT assessment of bone and cartilage was performed. When the feasibility of longitudinal imaging for tracking bony spurs over time is limited, analysis of joint diameter can serve as a simple approach to identify structural changes throughout the study period.

Neuropathic arthropathy represents a severe type of RPOA, a joint disease that progresses due to nerve damage, leading to a reduced ability to sense pain in the joint, as well as diminished joint proprioception (Chisholm and Gilchrist, 2011). To investigate symptomatic effects on weight bearing of the hindlimbs in our studies, we implemented an observer independent method. During in-life phase, static incapacitance tests revealed unloading of operated joints after surgery that was improved with 0.3 and 3 mg/kg anti-NGF compared to vehicle injection, but only during the first half of the experiment. In a previous publication, we showed steady improvements in incapacitance after systemic injections of anti-NGF (Gigout et al., 2022), however with a different time course and interventions. The subgroup of three rabbits receiving 3 mg/kg anti-NGF showed better performance in weight bearing compared to the remaining animals in this group. Considering these findings in conjunction with higher subchondral sclerosis and cartilage damage of this subgroup, this strengthens the hypothesis that analgesia-induced joint overloading has led to exacerbation of bone and cartilage damage.

A main limitation of this study was our establishment of a new method of 24/7-activity tracking during the course of the study (data analysis is ongoing and results will be published separately). Weight bearing measurements, which were assessed in parallel, are very sensitive to the rabbits’ body tension. During implementation of weight bearing in former studies, we discovered, that combining incapacitance with other events like subsequent blood sampling (on awake animals) or claw clipping resulted in stressed and tensed animals affecting, e.g., baseline values in healthy animals with increased scattering of data. For activity tracking, rabbits had to be caught out of the housing more often and changing their collars and trackers might have induced additional stress. As weight bearing only displays a snapshot in comparison to the duration of a long-term study, rabbits as prey animals might behave differently once placed in their housing pen and being undisturbed again (Miller and Leach, 2023). Therefore, additional symptomatic readouts like 24/7-activity data after treatment could provide more insights into this topic, as anti-NGF treatment reversed decreased activity levels resulting from instability surgery in a rat model, indicating the analgesic effects of anti-NGF (Brenneis et al., 2016).

Our study has demonstrated that administration of an anti-NGF antibody has negative effects on structural parameters like subchondral bone and cartilage in destabilized rabbit joints. These findings were particularly evident in individual animals, where high anti-NGF doses showed particularly good analgesic effects. On the other hand, slight improvement in pain-induced joint unloading could be observed. The combined consideration of structural and symptomatic readouts suggests that analgesic conditions probably exacerbate joint damage. These findings have the potential to provide a better understanding of the effects of systemic anti-NGF treatment and can therefore support the development of novel therapeutic interventions for OA patients to modulate chronic pain.

## Data Availability

The raw data supporting the conclusion of this article will be made available by the authors, without undue reservation.
