# Supplementary material for: Anti-NGF treatment worsens subchondral bone and cartilage measures while improving symptoms in floor-housed rabbits with osteoarthritis
Source: Front Physiol. 2023 Jun 26;14:1201328. doi: 10.3389/fphys.2023.1201328 (PMC10331818; doi:10.3389/fphys.2023.1201328)
Supplement: Supplementary file 1 [file DataSheet1.docx]

Supplementary Material

# Supplementary Figures and Tables

## Supplementary Figures


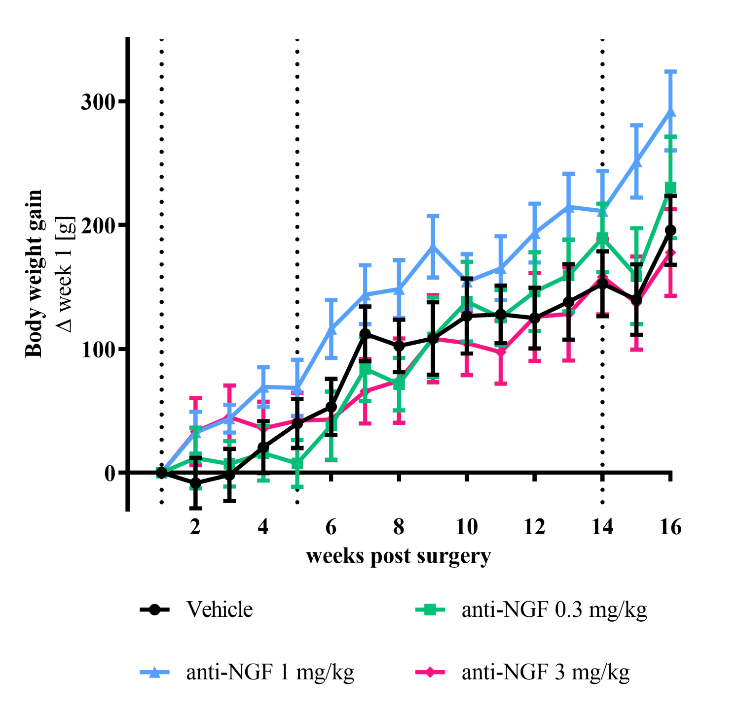


**Supplementary Figure 1.** Body weight gain over time; *n*=10-16; mean ± SEM; dotted black lines mark timepoint of injection of all groups; data passed Shaprio-Wilk test for normal distribution; no significant differences in 2way ANOVA with Tukey multiple comparison test.


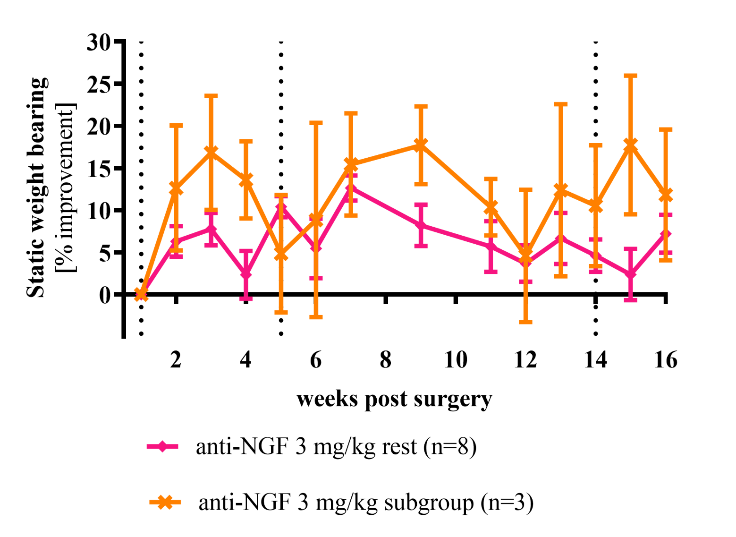


**Supplementary Figure 2.** Weight bearing time course of anti-NGF 3 mg/kg with subgroup; *n*=3-8; mean ± SEM; dotted black lines mark timepoint of injection of all groups; no outliers via ROUT method detected; data passed Shaprio-Wilk test for normal distribution; no significant differences in multiple unpaired t-tests.


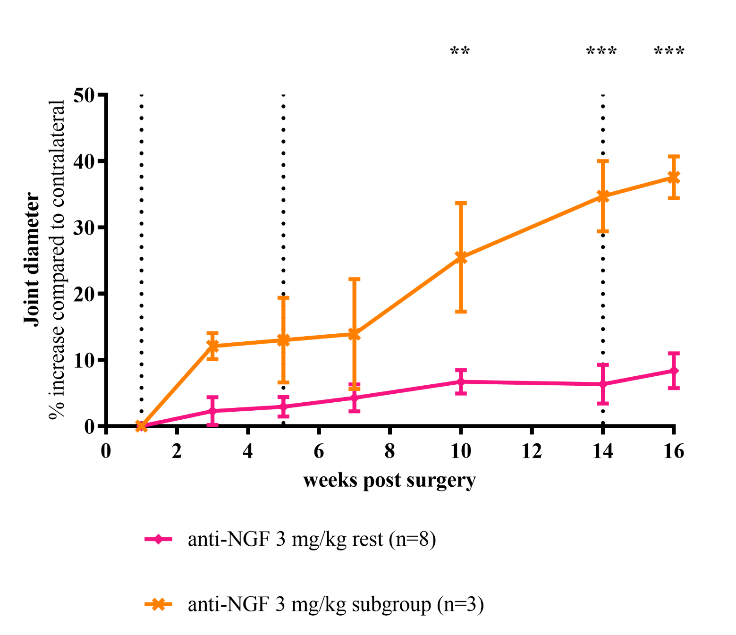


**Supplementary Figure 3.** Time course of joint diameter of anti-NGF 3 mg/kg with subgroup; n=3-8; mean ± SEM; dotted black lines mark timepoint of injection of all groups; no outliers via ROUT method detected; data passed Shaprio-Wilk test for normal distribution; results of multiple unpaired t-tests: week 10 ***p*<0.01; week 14 ****p*<0.001; week 16 ****p*<0.001.
